# A mobile interactive cognitive self-assessment scale for screening cognitive impairment due to Alzheimer’s disease

**DOI:** 10.1093/ageing/afae293

**Published:** 2025-01-25

**Authors:** Kexin Xie, Juan Huang, Ting Chen, Dan Li, Tianxinyu Xia, Min Chu, Yue Cui, Mei Tang, Dantao Peng, Jingtong Wang, Jianling Liu, Xiaojuan Chen, Weiwei Cui, Li Liu, Yingtao Wang, Jianbing Liu, Fang Li, Liyong Wu

**Affiliations:** Department of Neurology, Xuanwu Hospital, Capital Medical University, Beijing, China; Department of Neurology, FuXing Hospital, Capital Medical University, Beijing, China; Institute for Smart Ageing, Beijing Academy of Science and Technology, Beijing, China; Department of Neurology, Xuanwu Hospital, Capital Medical University, Beijing, China; Department of Neurology, Xuanwu Hospital, Capital Medical University, Beijing, China; Department of Neurology, Xuanwu Hospital, Capital Medical University, Beijing, China; Department of Neurology, Xuanwu Hospital, Capital Medical University, Beijing, China; Department of Geriatric Medicine, Beijing Friendship Hospital Affiliated to Capital Medical University, Beijing, China; Department of Neurology, China-Japan Friendship Hospital, Ministry of Health, Beijing, China; Department of Gerontology, Peking University People’s Hospital, Beijing, China; Home Care Center for Senior People, Beijing Yangfangdian Hospital, Beijing, China; Beijing Active Ageing and Smart Service Technology Co. Ltd., Beijing, China; Beijing Active Ageing and Smart Service Technology Co. Ltd., Beijing, China; Department of Neurology, Xuanwu Hospital, Capital Medical University, Beijing, China; Department of Neurology, Xuanwu Hospital, Capital Medical University, Beijing, China; Institute for Smart Ageing, Beijing Academy of Science and Technology, Beijing, China; Department of Neurology, FuXing Hospital, Capital Medical University, Beijing, China; Department of Neurology, Xuanwu Hospital, Capital Medical University, Beijing, China; National Clinical Research Center for Geriatric Diseases, Beijing, China

**Keywords:** cognitive impairment, Alzheimer’s disease, diagnostic accuracy, mobile cognitive assessment scale, screening measure, validation, older people

## Abstract

**Background:**

A mobile cognition scale for community screening in cognitive impairment with rigorous validation is in paucity. We aimed to develop a digital scale that overcame low education for community screening for mild cognitive impairment (MCI) due to Alzheimer’s disease (AD) and AD.

**Methods:**

A mobile cognitive self-assessment scale (CogSAS) was designed through the Delphi process, which is feasible for the older population with low education. In Phase 1, 518 clinically diagnosed participants were subjected to optimise the items. In Phase 2, the scale was validated in 358 participants with cognitively unimpaired and 396 participants of clinically diagnosed MCI and dementia for reliability, validity and diagnostic accuracy. In Phase 3, specificity and sensitivity were tested for biologically diagnosed participants of 38 with cognitively unimpaired and 45 with MCI and dementia due to AD according to the amyloid, tau, neurodegeneration classification system.

**Results:**

The CogSAS was a three-task mobile scale testing memory and executive function. In Phase 2, the internal consistency was 0.81, and the test–retest reliability was 0.82. The construct validity was 0.74, and the criterion validity was 0.77. The sensitivity and specificity for discriminating clinically diagnosed participants with MCI and dementia from cognitively unimpaired were 0.90 and 0.67, respectively. For discriminating biologically diagnosed MCI and dementia due to AD from cognitively unimpaired, the sensitivity and specificity were 1.00 and 0.78, respectively.

**Conclusions:**

The CogSAS has good reliability, validity and feasibility, showing a high sensitivity and specificity both in the community and the clinic, identifying biologically diagnosed MCI and dementia due to AD.

## Key Points

- We developed an interactive digital cognitive self-assessment scale, cognitive self-assessment scale (CogSAS), for screening cognitive impairment with Alzheimer’s disease (AD) pathology.
- The development of CogSAS used Delphi process and item optimisation in participants from the clinic and community.
- The CogSAS showed good reliability and validity in discriminating participants with clinically diagnosed participants with mild cognitive impairment (MCI) and dementia from those who are cognitively unimpaired.
- The CogSAS showed good diagnostic accuracy in biologically diagnosed MCI and dementia due to AD.
- The CogSAS can be further applied in community screening of clinically diagnosed MCI and dementia, and biologically diagnosed MCI and dementia due to AD.

## Background

Alzheimer’s disease (AD) is the leading cause of dementia in the general community [1]. Early diagnosis of cognitive impairment due to AD, including mild cognitive impairment (MCI) and dementia, improves functional preservation and primary care [2]. To achieve early diagnosis through community screenings, an efficient and self-administered cognitive scale would be optimal. However, current digital scales cannot provide satisfactory diagnostic accuracy and feasibility in screening for cognitive impairment due to AD with rigorous validation in large populations, and are therefore impractical for community screening.

Numerous attempts have been made to develop a novel digital cognitive assessment to solve this problem [3]. To address the inconvenience of labour-consuming traditional pen-and-paper tests, some have been converted to digital versions and validated in the memory clinic [4]. However, these computerised scales, such as the Cogstate and Montreal Cognitive Assessment (MoCA) [5, 6], have shown weak validity and inconsistent sensitivity in discriminating AD. Only Computer Assessment of Mild Cognitive Impairment (CAMCI) has been validated in a large population cohort for MCI, with a sensitivity of 86% and specificity of 94%. In contrast, a digital cognitive assessment for screening AD in a large population cohort remains vacant [7]. Most of the scales still require supervision and explanation by neurologists [8, 9]. These problems have affected the credibility and feasibility of revised scales for screening for MCI and dementia due to AD. To improve their feasibility and credibility, novel digital cognition tests using mobile applications have also been developed [9–11]. However, the validation for large populations of patients with MCI and dementia due to AD has been poor. In addition, the feasibility of existing mobile scales was influenced by education level. In China’s ageing population, 29.6% of them are illiterate, and 41.5% of them are primary school graduates [12]. Low education levels limit the use of mobile applications while existing scales require certain abilities to use mobile phones. Therefore, a mobile cognitive assessment scale, with improved performance in screening MCI and dementia due to AD and is compatible for elder population with low education, is urgently needed.

Thus, we developed an interactive cognitive self-assessment scale (CogSAS) that covers important domains of impairment in AD patients and was designed for assessing cognitive impairment with AD pathology. We optimised the scale with Delphi process and large population. To test the ability of the scale, we validated the sensitivity, specificity, reliability and validity within participants with clinically diagnosed MCI and dementia, and participants with biologically diagnosed MCI and dementia due to AD.

## Methods

**Figure 1:**
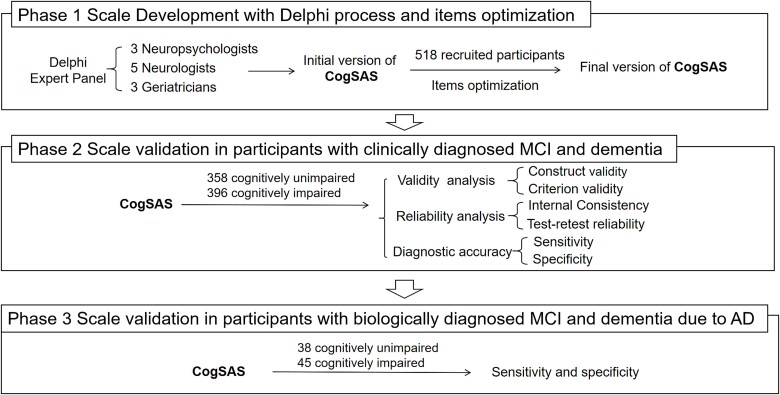
Details of CogSAS development and validation.

The flowchart in Figure 1 shows a summary of the three phases in which the CogSAS was developed and validated. The study was conducted from 2017 to 2023, from scale development to validation analysis, and included six medical centres and communities in Beijing, China.

### Procedures

#### Scale development

##### Design of the initial CogSAS version

Our purpose was to create a cognitive assessment scale according to part of the most characteristic cognition domains in AD pathology, including the memory and executive functions. The scale was designed as a mini-application for touch panels, smartphones and personal computers that works on both the WeChat app and the website. The scale was designed to be used alone or accompanied by caregivers, including social workers and family members. Immediate feedback of the results was presented at the end of the test. The scale was designed to be particularly age-friendly. First, the scale was adapted for the touchscreen to enhance practicality. Second, to be age-friendly, we applied speech recognition to provide instructions for the memory task procedure. By using voice instead of words, the influence of education level on this scale was reduced compared to pen-and-paper tests. Two human–computer interaction technicians were recruited to provide technical support.

The Delphi technique was used to develop the initial version of the scale. The expert panel included three neuropsychologists, five neurologists and three geriatricians (Appendix 2). All the experts were invited on the basis of their academic credentials and experience in clinical practice specialising in dementia and geriatrics. All the experts were willing to work through the entire scale development process. Three Delphi rounds were conducted to form an initial version of the CogSAS (Appendix 1: Delphi process).

##### Item optimisation

To further improve the credibility of the scale, item optimisation was conducted for the cohort after the Delphi process. The aim of item optimisation was to select applicable, sensitive items from the initial CogSAS version, adjust the scoring scheme and establish the final version. Item optimisation was conducted with clinically diagnosed MCI and dementia and cognitively unimpaired (CU) participants, applying receiver operating characteristic (ROC) analysis.

Scale item optimisation was conducted with 518 participants recruited from communities. Participants, all of whom were older than 55 years, were classified as CU or cognitively impaired, the latter of which included dementia and MCI. Dementia was diagnosed according to the Diagnostic and Statistical Manual of Mental Disorders, Fifth Edition (DSM-V) criteria [13], and MCI was diagnosed according to the Peterson criteria [14]. The Clinical Dementia Rating (CDR) was also used in the diagnosis of MCI and dementia. The eligibility criteria for participants without cognitive impairment were no reports of cognitively impaired performance and a CDR score of 0. Participants who were unable to cooperate on the neuropsychological examinations and digital scale were excluded. Here, we defined clinically diagnosed MCI and dementia as those participants who were diagnosed by the clinical interview, physical examinations and psychometric instruments, according to the inclusion criteria mentioned above. After the optimisation (Appendix 1: item optimisation, Appendix 3, Appendix 4), the scale score was also adjusted to increase item sensitivity. The self-reports section was deleted, which decreased the operation time from 30 to 15 min.

#### Scale validation in participants with clinically diagnosed MCI and dementia

In Phase 2, scale validation aimed to test the validity and reliability of the final CogSAS version. Validation was conducted with participants who were clinically diagnosed with MCI and dementia and those who were CU.

In addition to those who participated in item optimisation, 754 participants were recruited from the communities and six medical centres were enrolled in Phase 2. The inclusion criteria, diagnostic criteria, exclusion criteria, clinical data collection and neuropsychological assessments were the same as those described above for Phase 1.

Participants were asked to complete the final version of the CogSAS. Internal consistency, test–retest reliability, construct validity and criterion validity were used to test the reliability and validity of the CogSAS. A total of 70 participants were retested in the clinic 2–4 weeks after the initial tests for test–retest reliability. The ROC curve was also used to assess the diagnostic ability for identifying participants who were clinically diagnosed with MCI and dementia. A cut-off value for the total score was calculated based on the discrimination between participants who were CU and those who were clinically diagnosed with MCI and dementia. The sensitivity, specificity and likelihood ratio were also assessed.

#### Scale validation in participants with biologically diagnosed MCI and dementia due to AD

Given that the CogSAS was designed to screen for cognitive impairment due to AD, participants with biologically diagnosed MCI and dementia due to AD were also recruited to test their ability to discriminate cognitive impairment due to AD.

Phase 2 participants who completed comprehensive clinical data collection, magnetic resonance imaging (MRI) and lumbar puncture were enrolled in Phase 3. A total of 83 participants were enrolled with a diagnosis supported by the amyloid, tau, neurodegeneration (ATN) classification system [15, 16]. All participants were classified based on the Phase 2 inclusion criteria. For those who were classified as CU, ‘A−T−N−’ was tested; for those who were classified as MCI and dementia, ‘A+T+N+’ was tested. Here, we defined participants that are MCI and dementia with ‘A+T+N+’ as biologically diagnosed MCI and dementia due to AD, and CU participants with ‘A−T−N−’ as biologically diagnosed cognitively unimpaired.

After the MRI scan, a clinical diagnosis-blind medial temporal lobe atrophy (MTA) score was assigned independently by two experienced neurologists according to the standard criteria [17, 18]. A lumbar puncture was also performed within 1 week after the MRI. Cerebrospinal fluid Aβ and tau protein levels were assessed by a qualified laboratory. Detailed clinical data were collected, and the ATN classification system was used [15], where ‘A+’ was defined as an Aβ42/40 ratio <0.1, ‘T+’ was defined as a decreased phospho-tau value (<35.84 pg/ml) and ‘N+’ was defined as an MTA score ≥1.

The sensitivity, specificity and likelihood were also re-examined and compared with the diagnostic accuracy in Phase 2.

#### Statistical analysis

The data were analysed using the Statistical Package for the Social Sciences (PC version 23.0; IBM, Inc., New York, USA) and GraphPad Prism software (version 8.3.0; GraphPad Software, Inc., La Jolla, CA). Between-group differences in age, years of education and cognitive scores were assessed by Student’s *t*-test or analysis of variance with Tukey’s *post hoc* test. Sex differences were assessed using a chi-square test.

Reliability is assessed by test–retest reliability and internal consistency [19]. Validity is assessed by construct validity and criterion validity [20]. ROC curves were plotted to visualise sensitivity and specificity and to determine cut-offs. The area under the curve (AUC) was used to assess diagnostic accuracy. Youden’s index was used to select optimal cut-off values. *P* = 0.05 was considered to indicate statistical significance (Appendix 1: Statistical analysis).

## Results

### Scale development

**Figure 2:**
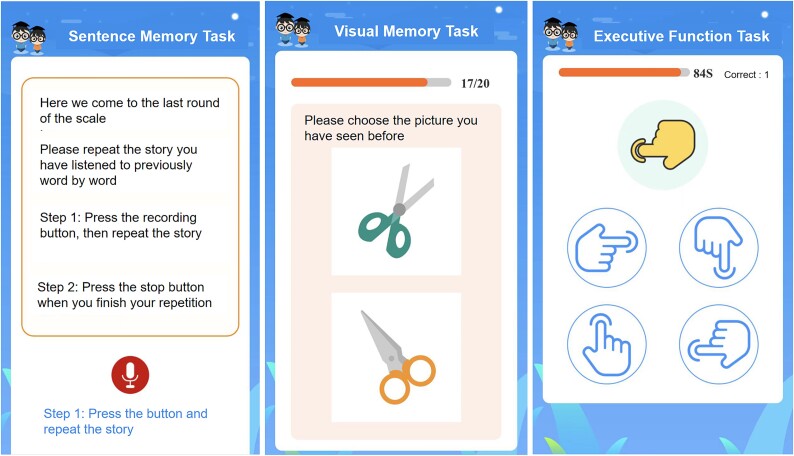
Schematic of the final digital CogSAS version. The instructions are shown as translations.

The final CogSAS version consists of three tasks, completed within 15 min, with a total scale score ranging from 0 to 64 (Figure 2). The task details are as follows:

Task 1—The sentence memory task (SEN): The SEN is a sentence repetition task where the participant repeats a short story sentence following a voice prompt. Each sentence contains essential verbal elements and scored up to 8 points. Participants repeat the sentence immediately after hearing it for the first time. After the first repetition, the participant listened to the sentence again and was asked to repeat the sentence a second time. Approximately 10 min later, at the end of the full scale, the participant was asked to recall the same sentence a third time. Each trial is scored according to the number of elements recalled, with a range of 0–8 points.

Task 2—Visual memory task (VIS): The VIS consists of 20 colour pictures selected base on the Chinese cultural background (Material S4). These pictures (set 1) are shown consecutively on screen for 10 s each. The participant was asked to remember each picture (encoding phase). The recognition task was administered 5 min later, and a target picture and a distractor were included (set 2). The distractor is designed to resemble the target picture in format or colour. The time limit for each item was 10 s. Each correct response is worth 1 point on a scale from 0 to 20, with higher scores indicating better recall.

Task 3—The executive function task (EXE): The EXE assesses responses to conflicting instructions. Participants viewed four finger shapes pointing in a different direction (response key), followed by an instruction key. If the instruction finger was white, the participant pressed the corresponding response key to the instruction key. If the instruction finger was yellow, the participant pressed the opposite response key to the instruction key. The instruction fingers were presented in random order at 10-s intervals. Participants completed a practice round before a 90-s task. Scores range from 0 to 20 based on the total number of correct responses.

### Scale validity and reliability analyses

Validity and reliability analyses were also conducted for the participants in Phase 2; 358 of whom were classified as CU and 396 of whom were clinically diagnosed MCI and dementia. There were no significant age or sex differences between the clinically diagnosed MCI and dementia and CU participants. Those who were clinically diagnosed MCI and dementia had fewer years of education (*P* < 0.05), and both the Mini Mental State Examination (MMSE) and MoCA scores were lower among participants who were clinically diagnosed MCI and dementia (*P* < 0.05) (Table 1).

**Table 1:** Demographics of Phase 2 participants with clinically diagnosed MCI and dementia and cognitively unimpaired

	Cognitively unimpaired	Clinically diagnosed MCI and dementia	*P* value
Number	358	396	
Age	68.49 ± 6.62	69.52 ± 7.89	0.052
Sex (male/female)	173/185	168/228	0.121
Years of education	12.90 ± 3.69	10.77 ± 4.27	0.000^*^
MMSE	28.63 ± 1.38	22.84 ± 5.56	0.000^*^
MoCA	25.67 ± 2.68	17.19 ± 5.67	0.000^*^

^*^ *P* < 0.05

#### Reliability analysis

The internal consistency of the CogSAS was assessed using Cronbach’s alpha coefficient to determine reliability. The Cronbach’s alpha coefficient was 0.81, indicating the internal consistency of the scale.

Test–retest reliability was assessed using Pearson’s correlation, and the coefficient was 0.82 (*P* < 0.001) between the first test and the repeated test.

#### Validity analysis

Construct validity was assessed using the KMO (0.74) and Bartlett’s tests (0.00), which verified the validity of the scale.

Criterion validity was verified by comparing the scale with the MMSE and MoCA as the ‘gold standards’. The CogSAS total score was positively correlated with both the MMSE (*R* = 0.77, *P* < 0.001) and the MoCA (*R* = 0.76, *P* < 0.001).

### Sensitivity and specificity analyses

Sensitivity and specificity were both tested by ROC curves for patients in Phase 2 and Phase 3.

Of the participants in Phase 3, 38 were classified as CU, and 45 were biologically diagnosed with MCI and dementia due to AD based on the ATN classification system. No significant differences were found in age or sex between the biologically diagnosed MCI and dementia due to AD and CU participants. A lower level of education and lower MMSE and MoCA scores were found in the participants with biologically diagnosed MCI and dementia due to AD (*P* < 0.05) (Table 2).

**Table 2:** Demographics of Phase 3 participants with biologically diagnosed MCI and dementia due to AD and are cognitively unimpaired

	Cognitively unimpaired	Biologically diagnosed MCI and dementia due to AD	*P* value
Number	38	45	
Age	65.68 ± 6.30	66.36 ± 7.59	0.666
Sex (male/female)	18/20	19/26	0.664
Years of education	11.79 ± 4.21	9.67 ± 4.24	0.025^*^
MMSE	28.42 ± 1.00	17.38 ± 6.70	0.000^*^
MoCA	25.18 ± 2.25	13.20 ± 6.86	0.000^*^

^*^ *P* < 0.05.

#### Sensitivity and specificity in discriminating participants with clinically diagnosed MCI and dementia

Among participants in Phase 2, the ROC curve showed an optimal cut-off point of 38.7 with an AUC of 0.86, a sensitivity of 0.90 and a specificity of 0.67. The likelihood ratio was 2.73, and the negative likelihood ratio was 0.15 (Table 3 and Appendix 7). Using ROC analysis, the optimal cut-off, sensitivity and specificity for each subtest were also evaluated (Appendix 5 and Appendix 8).

**Table 3:** Comparison of diagnostic accuracy between participants with clinically diagnosed MCI and dementia and biologically diagnosed MCI and dementia due to AD

	Phase 2: Diagnostic accuracy in participants with clinically diagnosed MCI and dementia	Phase 3: Diagnostic accuracy in participants with biologically diagnosed MCI and dementia due to AD
Number	754	83
Sensitivity	0.90	1.00
Specificity	0.67	0.78
Positive likelihood ratio	2.73	4.54
Negative likelihood ratio	0.15	0.00

#### Sensitivity and specificity in assessing participants with biologically diagnosed MCI and dementia due to AD

The CogSAS was subsequently used to classify participants in Phase 3 with biologically diagnosed MCI and dementia due to AD or CU based on the cut-off value. The ROC curve showed a sensitivity of 1.00, a specificity of 0.78 and an AUC of 0.93, outperforming those of participants from Phase 2. The likelihood ratio was 4.54, and the negative likelihood ratio was 0.00 (Table 3 and Appendix 7). The optimal cut-off, sensitivity and specificity for each subtest were also calculated (Appendix 6 and Appendix 9).

For those with biologically diagnosed MCI and dementia due to AD, the cut-off point was used to classify the participants according to their ATN classification system-based diagnosis. In Phase 3, 85.5% of participants were correctly classified by the CogSAS.

## Discussion

This study aimed to develop and validate the CogSAS, a cognitive assessment tool tailored for both community and clinical populations, focusing on part of cognitive domains affected in AD. Compared with previous mobile scales, the CogSAS is suitable for large populations and has good validity, reliability and feasibility. Its high diagnostic accuracy for biologically diagnosed MCI and dementia due to AD highlights its potential for early detection of AD-related cognitive impairment.

The development of CogSAS followed a rigorous process, including the use of Delphi methods to ensure expert input, as well as iterative optimisation with large population samples, enhancing credibility compared to scales solely refined by expert review panels [21]. Unlike in the opinion of experts, database optimisation of the CogSAS was more credible. Furthermore, we recruited participants with AD pathology, which improved the credibility of identifying biologically diagnosed MCI and dementia due to AD. Additionally, we used speech recognition and other human–computer interaction techniques to reduce the influence of education level. Compared to text, using voice prompting and speech recognition could broaden the application of this tool in community screening, regardless of education level. We have also made efforts in Mandarin accent recognition to promote its application in more regions of China.

Through the development process, the CogSAS was validated and showed relatively good validity, reliability and high diagnostic accuracy. The Cronbach’s alpha and the test–retest reliability are within the acceptable range (0.70–0.90) [22]. The validity of previous digital scales was 0.77 for the Computer-Administered Neuropsychological Screen for Mild Cognitive Impairment (CANS-MCI), while some others were not well validated [23]. Furthermore, construct validity and criterion validity of the scale reached acceptable validity [24]. Previous digital scales showed variable criterion validity (0.21–0.62) and construct validity (0.76–0.77) [23, 25, 26]. These results suggest that the CogSAS is credible for identifying people with biologically diagnosed MCI and dementia due to AD.

Diagnostic accuracy was reflected in the sensitivity, specificity and likelihood ratio. An ideal screening scale should have high sensitivity to ensure follow-up diagnosis in vulnerable individuals [27], while moderate specificity is acceptable. The CogSAS showed high sensitivity (up to 1.00) and acceptable specificity, suggesting that it is an ideal tool for community screening. Compared with existing digital scales, the sensitivity of those varies from 0.41 to 1.00, and the specificity varies from 0.6 to 0.96 [4]. The CogSAS also showed acceptable likelihood ratios (positive likelihood 2.73–4.54, negative likelihood ratio 0.00–0.15) [28].

The CogSAS showed good performance in discriminating cognitive impairment from part of the AD pathology, including memory and executive function. First, the scale was designed primarily for participants with cognitive impairment with AD pathology, using minimal assessments and feasible methods. Memory loss is a hallmark of AD, with an emphasis on the characteristic effect on episodic and working memory [29, 30]. Here, we used the sentence memory task to assess episodic memory and a contradicting direction test to assess working memory and executive function. Second, in assessing criterion validity, a high correlation coefficient was found with both the MMSE and the MoCA, which are well recognised for diagnosing MCI and dementia due to AD [31]. Finally, a higher sensitivity (1.00) was found for detecting cognitive impairment with AD pathology. Compared with the sensitivities of 0.7–0.9 for existing scales that discriminate cognitive impairment [4], the CogSAS showed higher sensitivity, suggesting that it may be accurate screening for biologically diagnosed MCI and dementia due to AD.

Despite its strengths, this study has limitations. First, the subgroups with biomarkers were relatively small; highlighting the need for replication in larger samples. Second, sensitivity and specificity were assessed only in the clinic; future studies should focus on longitudinal community monitoring and mass screening. Finally, the study recruited participants with MCI and mild to moderate AD, while subjective cognitive decline, which is characterised by a self-experienced persistent decline in cognition with normal performance in standardised cognitive tests [32], should be included with earlier intervention.

## Conclusion

A novel, interactive cognitive assessment scale, the CogSAS, demonstrated strong reliability, validity, feasibility and good diagnostic accuracy. It effectively discriminates cognitive impairment, especially in the presence of AD pathology. The CogSAS may improve mass screening for cognitive impairment, leading to earlier diagnosis.

## Supplementary Material

aa-24-0884-File004_afae293

## Research data transparency and availability:

The data underlying the current study are not publicly available due to privacy and ethical reasons. Please contact the main author for more information (cindyxkx97@163.com).

## Notes

Kexin Xie, Juan Huang, Ting Chen and Dan Li contributed equally as first authors

Jianbing Liu, Fang Li and Liyong Wu contributed equally as corresponding authors
