# Supplementary material for: A mobile interactive cognitive self-assessment scale for screening cognitive impairment due to Alzheimer’s disease
Source: Age Ageing. 2025 Jan 25;54(1):afae293. doi: 10.1093/ageing/afae293 (PMC11761740; doi:10.1093/ageing/afae293)
Supplement: aa-24-0884-File004_afae293 [file aa-24-0884-file004_afae293.pdf]

A mobile interactive cognitive self-assessment scale for screening cognitive impairment due to Alzheimer's disease

Supplementary Data

- Appendix 1 eMethod
- Appendix 2 Table S1 Composition of the expert panel
- Appendix 3 Table S2 Demographic data of phase 1 participants who are clinically diagnosed MCI and dementia and those with cognitively unimpaired
- Appendix 4 Table S3 Items optimization of initial CogSAS version
- Appendix 5 Table S4 The diagnostic accuracy of items in participants with clinically diagnosed MCI and dementia from phase 2
- Appendix 6 Table S5 The diagnostic accuracy of items in biologically diagnosed MCI and dementia due to AD from phase 3
- Appendix 7 Figure S1 Receiver operating characteristic curve of the final CogSAS version
- Appendix 8 Figure S2 Receiver operating characteristic curve of items comparing participants with CU and clinically diagnosed MCI and dementia in phase 2
- Appendix 9 Figure S3 Receiver operating characteristic curve of items comparing participants with CU and biologically diagnosed MCI and dementia due to AD in phase 3

Appendix 1: eMethod

Delphi process

The expert panel was asked to develop the initial version of the CogSAS, which included memory and executive function tasks and three self-reported measures of psychological status and activities of daily living. Reviews of original research papers and literature reviews were also conducted to form the questionnaires for item selection. As for the items included, aside from the cognition tasks, we included self-reports scales including questions for cognition status self-report (based on Subjective Cognitive Decline Questionnaire (SCD-Q)), questions for emotion status self-report (based on Patient Health Questionnaire (PHQ-9)) and caregiver report task (based on Informant Questionnaire on Cognitive Decline in the Elderly (IQCODE)). SCD-Q is a questionnaire that used to quantify SCD. The scale contains two parts including MyCog, which is answered by the subject, and TheirCog, which includes the same questions as MyCog but are answered by the informant or caregiver. The questions from SCD-Q could detected SCD patients from cognitive normal.[1] PHQ-9 is a 9-items questionnaire designed to screen depression.[2] IQCODE is a questionnaire that designed for dementia and is filled out by a relative or other caregivers. The scale contains everyday situations and could discriminated dementia in variety of healthcare settings.[3] All the experts were sent to the online survey questionnaires. All the responses were assured of confidentiality throughout the whole process. The items were selected through three rounds of the Delphi process, with each round lasting more than 2 weeks. During round 1 (Material S1), the experts were asked to select the ideal items

for the scale and suggest additional items for inclusion in round 2. Reasons and amendments were also requested from those who disagreed. During round 2 (Material S2), in addition to item selection, the experts were asked to present the scoring rules for each item. A further round (third round) was carried out for the final selections of items and the establishment of the scoring rules (Material S3). In all three rounds of the Delphi process, the response rate of the expert panel was 100%. Items with more than 70% agreement were accepted. After all three Delphi rounds, offline discussions were held to review the results of the Delphi survey. The results of each Delphi process are presented (Additional file 3 and Additional file 4). All members of the expert panel participated in the discussion and decided on the initial version of the CogSAS. The initial version of the CogSAS was approved by all members of the expert panel.

Material S1 results of the round 1 Delphi survey

(Items in red were lacking agreement)

Item	Agree, N(%)	Disagree, N(%)
Verbal memory task		
Memorize words or a sentence and then recall the words or the sentence	11(100%)	0(0%)
Task design:<br>Answers (in Chinese): Face (面孔)、Velvet (丝绸)、School (学校)、Daisy (菊花)、Red (红色)<br>Distractor (in Chinese): Facial expression (表情)、Cotton (棉纱)、Hospital (医院)、Peony (牡丹)、Blue(蓝色)	4(36.4%)	7(63.6%)
Visual memory task		
Memorize pictures then recognize after a period of time	10(90.9%)	1(9.1%)
Task design:	3(27.3%)	8(72.7%)

Memorizing picture: 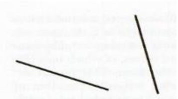<br><br>Recognizing picture: 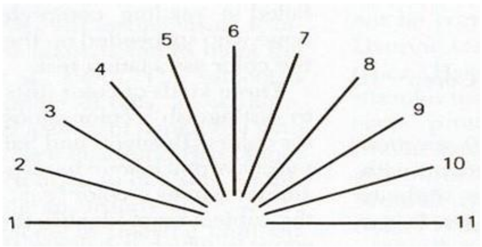<br>Select the number of lines that shown before		
Language task		
Choose the right picture according to the voice description	5(45.5%)	6(54.5%)
Task design:<br>Description: An earthworm is crawling through grassland<br>Pictures:<br>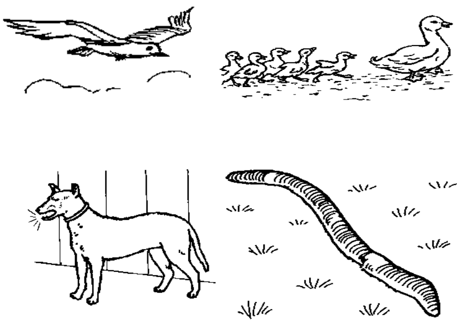<br>Choose the right picture according to the description	4(36.4%)	7(63.6%)
Executive function task		
Use “Go/No-go” task	10(90.9%)	1(9.1%)
Task design:<br>When the number “1” shows, press the button on the screen<br>When the number “2” shows, don’t press the button on the screen<br>The number will automatically change with each number lasts 5 seconds.<br>The order of the number is “1-2-1-2-1-1-2-2-1-1”	6(54.5%)	5(45.5%)
Self-report task		
Including cognition status self-report and emotion status self-report	11(100%)	0(0%)
Questions for cognition status self-report (based on SCD-Q)[4]		
All the questions above are evaluated based on the status of the past two years		
Having difficulty in remembering telephone numbers	9(81.8%)	2(18.2%)
Having difficulty in finding personal stuff (mobile phones, keys,	10(90.9%)	1(9.1%)

glasses etc.)		
Having difficulty retelling the story of a movie	9(81.8%)	2(18.2%)
Having difficulty retelling the story of a book	9(81.8%)	2(18.2%)
Having difficulty visiting doctors on time	5(45.5%)	6(54.5%)
Having difficulty recalling recent events	9(81.8%)	2(18.2%)
Having difficulty recalling recent news	8(72.7%)	3(27.3%)
Having difficulty remembering the shopping list	8(72.7%)	3(27.3%)
Having difficulty calculating when paying at a shop	4(36.4%)	7(63.6%)
Having difficulty continuing long conversations	5(45.5%)	6(54.5%)
Having difficulty recalling celebrities' names	8(72.7%)	3(27.3%)
Having difficulty recalling familiar people's names	8(72.7%)	3(27.3%)
Having difficulty remembering the latest news	7(63.6%)	4(36.4%)
Having difficulty remembering the names of locations	8(72.7%)	3(27.3%)
Having occasions when the words are on the tip of your tongue	9(81.8%)	2(18.2%)
Needing pencil and paper to help memorizing	3(27.3%)	8(72.7%)
Having difficulty concentrating	4(36.4%)	7(63.6%)
Having difficulty planning uncommon events in daily life (travel, parties etc.)	2(18.2%)	9(81.8%)
Having difficulty remembering places that have recently visited	8(72.7%)	3(27.3%)
Having difficulty using electronic products	8(72.7%)	3(27.3%)
Having difficulty trying new stuff	2(18.2%)	9(81.8%)
Having difficulty starting conversations	8(72.7%)	3(27.3%)
Having difficulty doing mental math	2(18.2%)	9(81.8%)
Having difficulty multitasking	1(9.1%)	10(90.9%)
Questions for emotion status self-report (based on PHQ-9)[5]		
Little interest or pleasure in doing things	10(90.9%)	1(9.1%)
Feeling down, depressed or hopeless	11(100%)	0(0%)
Trouble falling asleep, staying asleep, or sleeping too much	8(72.7%)	3(27.3%)
Feeling tired or having little energy	5(45.5%)	6(54.5%)
Poor appetite or overeating	9(81.8%)	2(18.2%)
Feeling bad about yourself - or that you're a failure or have let yourself or your family down	9(81.8%)	2(18.2%)
Trouble concentrating on things, such as reading the newspaper or watching television	7(63.6%)	4(36.4%)
Moving or speaking so slowly that other people could have noticed. Or, the opposite -being so fidgety or restless that you have been moving around a lot more than usual	10(90.9%)	1(9.1%)

Thoughts that you would be better off dead or of hurting yourself in some way	8(72.7%)	3(27.3%)
Caregiver report task (based on IQCODE)[6]		
Remembering things about family and friends, eg, occupations, birthdays, addresses	6(54.5%)	5(45.5%)
Remembering things that have happened recently	8(72.7%)	3(27.3%)
Recalling conversations a few days later	8(72.7%)	3(27.3%)
Remembering his/her address and telephone number	10(90.9%)	1(9.1%)
Remembering what day and month it is	8(72.7%)	3(27.3%)
Remembering where things are usually kept	10(90.9%)	1(9.1%)
Remembering where to find things which have been put in a different place from usual	9(81.8%)	2(18.2%)
Knowing how to work familiar machines around the house	8(72.7%)	3(27.3%)
Learning to use a new gadget or machine around the house	6(54.5%)	5(45.5%)
Learning new things in general	8(72.7%)	3(27.3%)
Following a story in a book or on TV	9(81.8%)	2(18.2%)
Making decisions on everyday matters	8(72.7%)	3(27.3%)
Handling money for shopping	4(36.4%)	7(63.6%)
Handling financial matters, eg, the pension, dealing with the bank	8(72.7%)	3(27.3%)
Handling other everyday arithmetic problems, eg, knowing how much food to buy, knowing how long between visits from family or friends	9(81.8%)	2(18.2%)
Using his/her intelligence to understand what's going on and to reason things through	8(72.7%)	3(27.3%)

Comment summary of the round 1 Delphi survey

Reasons for disagreement in Verbal memory task

- The chosen words are inconsistent with the Chinese cultural background and level of education. A one-sentence story could be used which is familiar to the Chinese ageing population.

- Both immediate recall and delayed recall should be tested

- More answers and distractors should be included

Reasons for disagreement in Visual memory task

- The picture is too abstract

- Pictures of familiar items or patterns should be given, which is familiar to Chinese culture and

regardless of level of education.

- More pictures should be included

- Less distractors should be provided

Reasons for disagreement in Language task

- It is not essential

-It could be replaced by verbal memory tasks by remembering a story

Reasons for disagreement in Executive function task

- More specific pictures could be used as instruction instead of numbers, which is easier to understand.

Reasons for disagreement in Self-report task

- The questions are redundant and time-consuming.

- It is unsuitable for a screening scale to include such an amount of questions. Limited and essential questions should be included.

- Some questions could be combined into one.

- Repeated questions are asked in cognition status and emotion status.

- Some questions are not applicable based on Chinese culture, like calculating when paying as cash is not used that often.

Reasons for disagreement in Caregiver report task

- Too many questions are included.

- Questions for caregivers should match the questions in self-reports to testify to participants' insight.

Suggestions for adding items

Verbal memory task:

-A one-sentence story: Last year, on the 7th of August, I went to see a circus show. There were goats walking on a tightrope, and monkeys riding bicycles. (去年八月七号，我去看了马戏表演，有山羊走钢丝，猴子骑单车)

Visual memory task:

- 20 to 30 pictures with stuff selected based on Chinese culture. The corresponding amount of distractor should be given.

Executive function task:

-Fingers that point in different directions could be used instead of numbers. When the instruction finger is white, choose the answer finger in the same direction. When the instruction finger is yellow, choose the answer finger in the opposite direction.

Material S2 results of the round 2 Delphi survey

(Items in red were lacking agreement)

Item	Agree, N(%)	Disagree, N(%)
Sentence memory task		
Task design:<br>Answers (in Chinese): Last year, on the 7th of August, I went to see a circus show. There were goats walking on a tightrope, monkeys riding bicycles. (去年八月七号，我去看了马戏表演，有山羊走钢丝，猴子骑单车)	8(72.7%)	3(27.3%)
Visual memory task		
Task design:<br>Memorize 20 to 30 pictures, and then recognize the pictures from distractor	7(63.6%)	4(36.4%)
Executive function task		
Task design:<br>When instruction finger is white, choose the answer finger with same direction. When the instruction finger is yellow, choose the answer finger with the opposite direction.	9(81.8%)	2(18.2%)
Self-report task		
Questions for cognition status self-report		
Having difficulty finding personal stuff (mobile phones, keys, glasses etc.)	10(90.9%)	1(9.1%)
Having difficulty in retelling the story of a movie or book	9(81.8%)	2(18.2%)
Having difficulty recalling recent events or news	9(81.8%)	2(18.2%)
Having difficulty shopping	8(72.7%)	3(27.3%)
Having difficulty in making conversations	9(81.8%)	2(18.2%)
Having difficulty recalling people’ s names (celebrity, familiar people etc)	8(72.7%)	3(27.3%)
Having difficulty remembering the names of locations	9(81.8%)	2(18.2%)
Having occasions when the words are on the tip of your tongue	11(100%)	0(0%)
Having difficulty using electronic products	9(81.8%)	2(18.2%)

Having difficulty in learning new things	10(90.9%)	1(9.1%)
Questions for emotion status self-report		
Feeling down, tired, depressed or hopeless, having little interest in doing things	10(90.9%)	1(9.1%)
Having problems eating and sleeping, including falling asleep, staying asleep, sleeping too much, eating too much or too less	9(81.8%)	2(18.2%)
Feeling bad about yourself - or that you're a failure or have let yourself or your family down, or would be better off dead or of hurting yourself in some way	9(81.8%)	2(18.2%)
Moving or speaking so slowly that other people could have noticed. Or, the opposite -being so fidgety or restless that you have been moving around a lot more than usual	10(90.9%)	1(9.1%)
Caregiver report task		
Remembering things or conversations that have happened recently	8(72.7%)	3(27.3%)
Remembering his/her address and telephone number	9(81.8%)	2(18.2%)
Remembering what day and month it is	11(100%)	0(0%)
Remembering where things are usually kept, or finding things which have been put in a different place from usual	10(90.9%)	1(9.1%)
Knowing how to work familiar machines around the house	9(81.8%)	2(18.2%)
Learning new things in general	10(90.9%)	1(9.1%)
Following a story in a book or on TV	10(90.9%)	1(9.1%)
Making decisions on everyday matters	8(72.7%)	3(27.3%)
Handling everyday arithmetic problems, eg, knowing how much food to buy, knowing how long between visits from family or friends, pension	9(81.8%)	2(18.2%)
Using his/her intelligence to understand what's going on and to reason things through	9(81.8%)	2(18.2%)

Comment summary results of the round 2 Delphi survey

Scoring rule for Sentence memory task:

- One point for every word

- One point for content words

Scoring rule for Visual memory task:

-One point for every picture

-Including 20 pictures are enough as 30 pictures are time-consuming

Scoring rule for Executive function task:

- One point for every correct answer.

Scoring rule for Self-report and Caregiver report task:

-One point for each “yes” answer to the question.

Material S3 results from the round 3 Delphi survey

(Items in red were lacking agreement)

Item	Agree, N(%)	Disagree, N(%)
Sentence memory task		
Scoring rules:<br>One point for every character, 25 points in total.	3(27.3%)	8(72.7%)
Scoring rules:<br>One point for content words, 8 points in total.	11(100%)	0(0%)
Visual memory task		
Task design:<br>Memorize 20 pictures, and then recognize the pictures from distractor<br>Detailed pictures are shown in Supplementary figures	9(81.8%)	2(18.2%)
Scoring rule:<br>One points for each correct answer, 20 points in total.	11(100%)	0(0%)
Executive function task		
Scoring rule:<br>One points for each correct answer	10(90.9%)	1(9.1%)
Self-report task		
Scoring rule<br>One points for each "yes" answer to the question	11(100%)	0(0%)
Caregiver report task		
Scoring rule<br>One points for each "yes" answer to the question	11(100%)	0(0%)

Item optimization

For all the 518 participants, they were interviewed by trained neuropsychologists who administered the Mini-Mental State Examination (MMSE) and MoCA within 24 hours. Participants were then asked to complete the initial CogSAS version without assistance via a touch panel, smartphone, or computer. All participants' performances were recorded and evaluated. The demographic and cognitive function data are shown (Table S2).

To select applicable items, sensitive items, operation times and scores were analysed. ROC analysis was used to optimize the scale. The item optimization criteria were as follows: 1) initial CogSAS version items were excluded if their area under the curve (AUC) was <0.6, and 2) initial CogSAS items that were completed by <70% of participants were also excluded. Additionally, we believe that the rather long operation time made the participants impatient. The cognition status of cognitively impaired participants may also impede their understanding of self-evaluation questions.

The scale score was also adjusted to increase item sensitivity. Regression and canonical discrimination analyses were also conducted to improve the screening methods used. Different assignments were designed to reach ideal validity and reliability. Other modifications, including adding time intervals between items, changing wording, and recording participants' years of education, were also assessed

Etiology of participants with clinically diagnosed cognitive impairment

In phase 1 and phase 2, we recruited clinically diagnosed MCI and dementia in our cohort. The inclusion criteria were listed in the Methods section. 83 participants in phase 3 who enrolled from phase 2 were biologically diagnosed with MCI and dementia due to AD. The aetiology of other participants includes 1)primary causes like neurodegenerative diseases, such as clinically diagnosed MCI and dementia for those patients who are unable to do the MRI scan and lumbar puncture; frontotemporal dementia; dementia with Lewy bodies, multiple system atrophy, progressive supranuclear palsy, Parkinson disease, cerebral amyloid angiopathy, and normal pressure hydrocephalus; 2)secondary causes like vascular dementia, autoimmune encephalitis; viral encephalitis, subacute combined degeneration, depression, and stroke; 3)unknown causes that we failed to get the final diagnosis of the patients.

Statistical analysis

Reliability refers to consistency when the scale is repeated under identical conditions and is assessed by test-retest reliability and internal consistency.[7] Test-retest reliability was used to assess the consistency of sum scores across time using Pearson's correlation coefficients.[8] The higher the correlation is, the greater the test-retest reliability.[9] Internal consistency was the degree to which the set of items varied relative to the sum of the scores and was measured using Cronbach's alpha coefficients. The acceptable threshold of the alpha coefficient was 0.7 for reliability.[7]

Validity is used to test whether the instrument indeed measures the latent dimension or aimed-evaluated construct, which is assessed by construct validity and criterion validity.[8] We constructed validity tests to determine whether the scale measures the intended construct by applying the Kaiser–Meyer–Olkin (KMO) test and Bartlett's test of factor analysis. The KMO test should be at least 0.5, and Bartlett's test showed significant results.[10] Criterion validity measures the relationship between the sum score and the criterion, typically referred to as the gold standard, which is assessed by Pearson's correlation coefficients. A higher correlation coefficient indicates greater validity.[8] ROC curves were plotted to visualize sensitivity and specificity and to determine cut-offs. The AUC was used to assess diagnostic accuracy. Youden's index was used to select optimal cut-off values. p=0.05 was considered to indicate statistical significance.

Appendix 2: Table S1 Composition of the expert panel

Name	Affiliation	Specialty	Years of Experience
Liyong Wu	Xuanwu Hospital	Neurologist	27
Fang Li	FuXing Hospital	Neurologist	25
Dantao Peng	China-Japan Friendship Hospital	Neurologist	30
Yue Cui	Xuanwu Hospital	Neurologist	7
Juan Huang	FuXing Hospital	Neurologist	7
Dan Li	Xuanwu Hospital	Neuropsychologist	25
Xiaojuan Chen	Beijing Active Ageing and Smart Service Technology Co. Ltd	Neuropsychologist	10
Weiwei Cui	Beijing Active Ageing and Smart Service Technology Co. Ltd	Neuropsychologist	14
Mei Tang	Beijing Friendship Hospital affiliated to Capital Medical University	Geriatric	29
Jingtong Wang	Peking University People's Hospital	Geriatric	22
Jianling Liu	Beijing Yangfangdian Hospital	Geriatric	24

Appendix 3: Table S2 Demographic data of phase 1 participants who are clinically diagnosed MCI and dementia and those with cognitively unimpaired

	Cognitively unimpaired	Clinically diagnosed MCI and dementia	*p* value
Number	240	278	
Age	67.58±6.75	67.28±7.48	0.573
Sex (Male/Female)	89/151	116/162	0.281
Years of education	10.99±3.82	8.80±4.12	0.014*

*$p < 0.05$

MCI, mild cognitive impairment

Appendix 4: Table S3 Items optimization of initial CogSAS version

		AUC	Sensitivity	Specificity	*p* value	Optimization Method
SEN	Before optimization	0.76	0.84	0.59	0.000*	Adjusting scoring points
	After optimization	0.81	0.78	0.70	0.000*	
VIS	Before optimization	0.69	0.44	0.89	0.000*	Decreased half of the figures following participants' feedback
	After optimization	0.76	0.93	0.52	0.000*	
EXE	Before optimization	0.60	0.42	0.83	0.000*	Adjusted scoring scheme
	After optimization	0.69	0.65	0.67	0.000*	
Self-reports	Before optimization	0.79	0.78	0.72	0.000*	Excluded for low complete percentage
	After optimization	/	/	/	/	
The total score points	Before optimization	/	/	/	/	Total score points after optimization
	After optimization	0.87	0.87	0.73	0.000*	

$^{*}p < 0.05$, SEN, sentence memory task; VIS, visual memory task; EXE, executive function task; AUC, area under curve

Appendix 5: Table S4 The diagnostic accuracy of items in clinically diagnosed MCI and dementia from phase 2

	SEN	VIS	EXE	Total score
Sensitivity	0.86	0.93	0.69	0.90
Specificity	0.65	0.52	0.71	0.67
AUC	0.83	0.78	0.74	0.86
Cutoff point	13.5	15.5	9.7	38.7

SEN, sentence memory task; VIS, visual memory task; EXE, executive function task; AUC, area under curve

Appendix 6: Table S5 The diagnostic accuracy of items in biologically diagnosed MCI and dementia due to AD from phase 3

	SEN	VIS	EXE	Total score
Sensitivity	0.90	0.87	0.92	1.00
Specificity	0.87	0.69	0.82	0.78
AUC	0.92	0.84	0.91	0.93

SEN, sentence memory task; VIS, visual memory task; EXE, executive function task; AUC, area under curve

Material S1 Pictures involve in Task 2

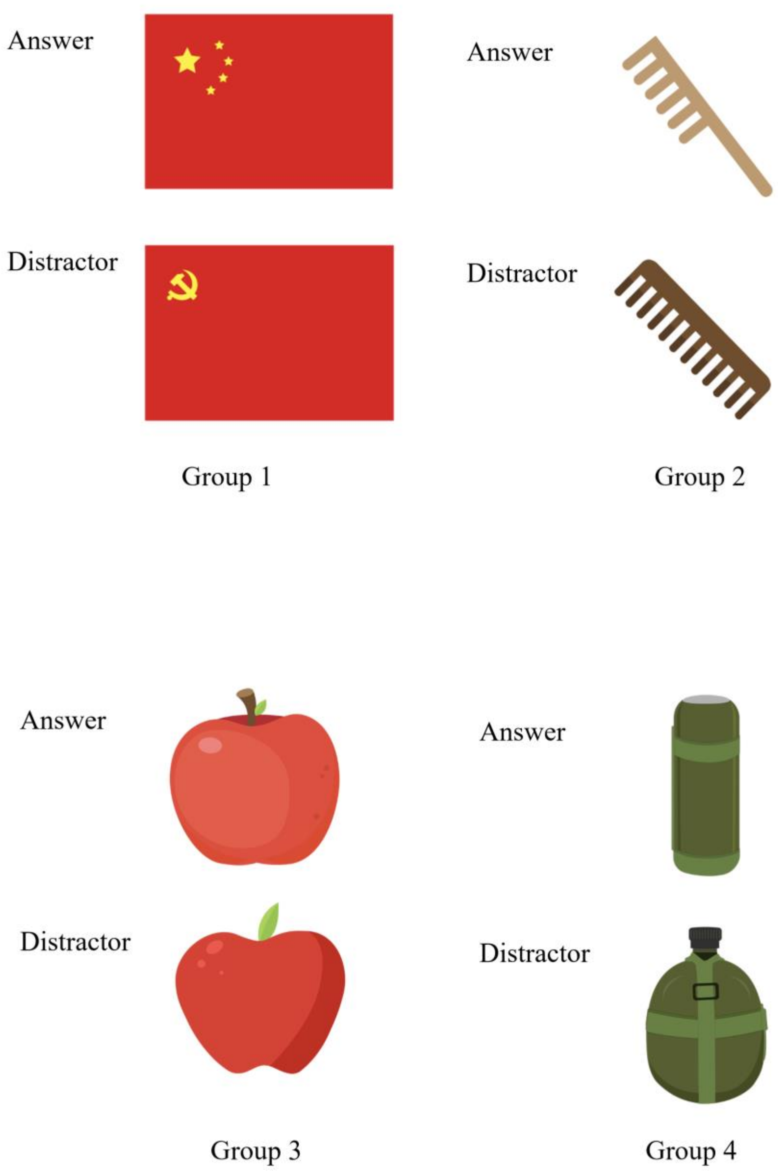

Answer

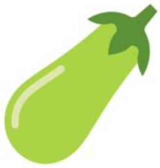

Distractor

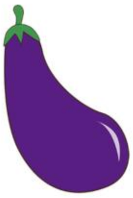

Group 5

Answer

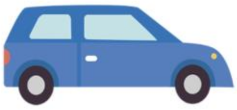

Distractor

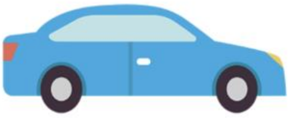

Group 6

Answer

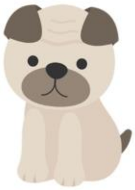

Distractor

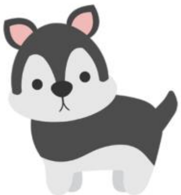

Group 7

Answer

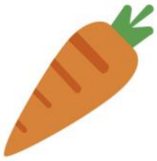

Distractor

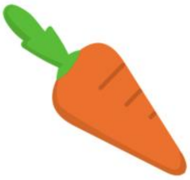

Group 8

Answer

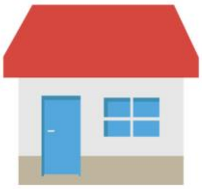

Distractor

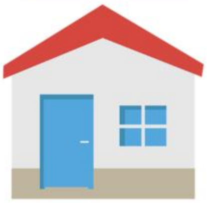

Group 9

Answer

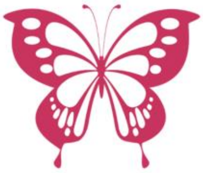

Distractor

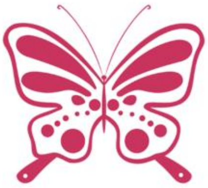

Group 10

Answer

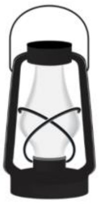

Distractor

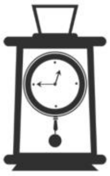

Group 11

Answer

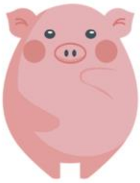

Distractor

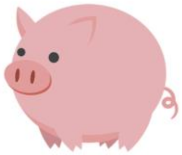

Group 12

Answer

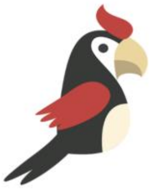

Distractor

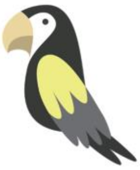

Group 13

Answer

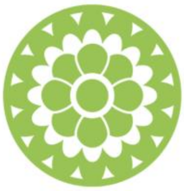

Distractor

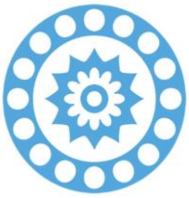

Group 14

Answer

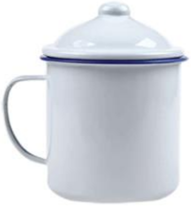

Distractor

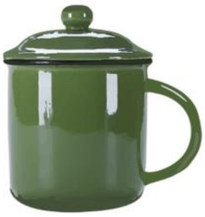

Group 15

Answer

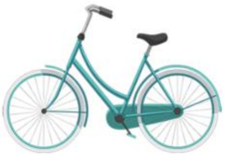

Distractor

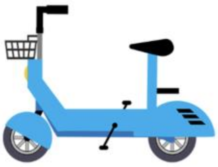

Group 16

Answer

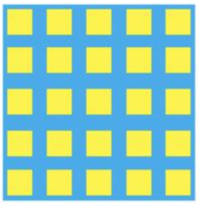

Distractor

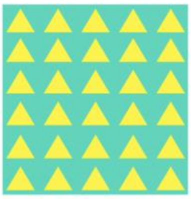

Group 17

Answer

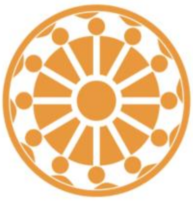

Distractor

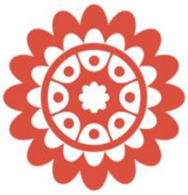

Group 18

Answer

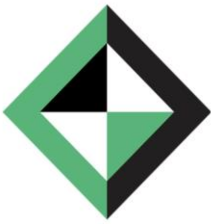

Distractor

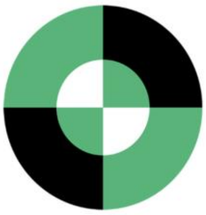

Group 19

Answer

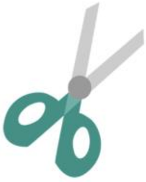

Distractor

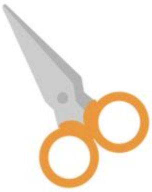

Group 20

Appendix 7: FIGURE S1 Receiver operating characteristic curve of the final CogSAS version

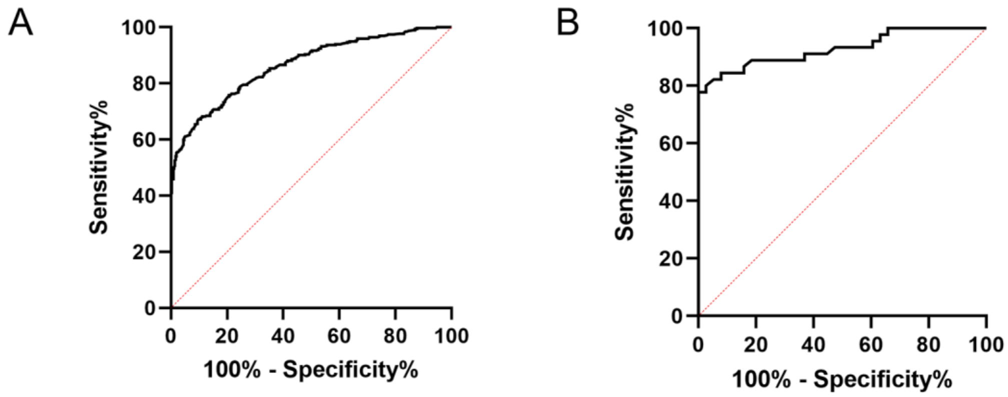

A, ROC curve of CogSAS discriminating participants clinically diagnosed MCI and dementia in phase 2; B, ROC curve of CogSAS discriminating participants with Mbiologically diagnosed MCI and dementia due to AD in phase 3

Appendix 8: FIGURE S2 Receiver operating characteristic curve of items comparing participants with CU and clinically diagnosed MCI and dementia in phase 2

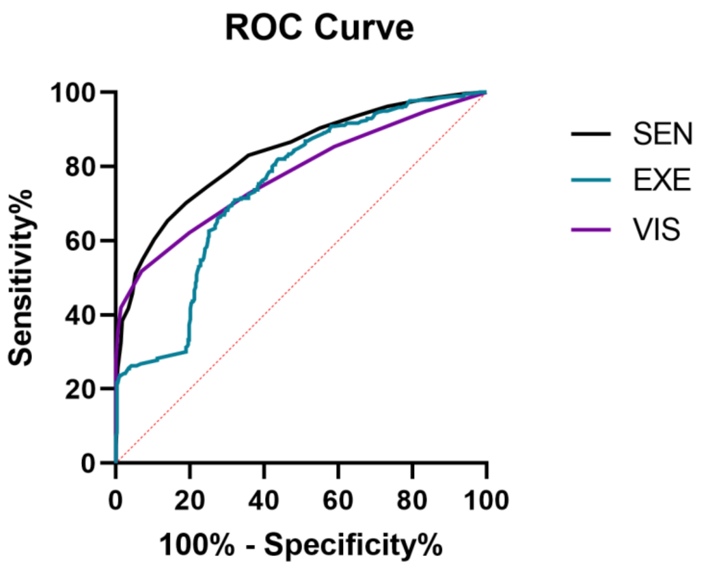

Appendix 9: FIGURE S3 Receiver operating characteristic curve of items comparing participants with CU and biologically diagnosed MCI and dementia due to AD in phase 3

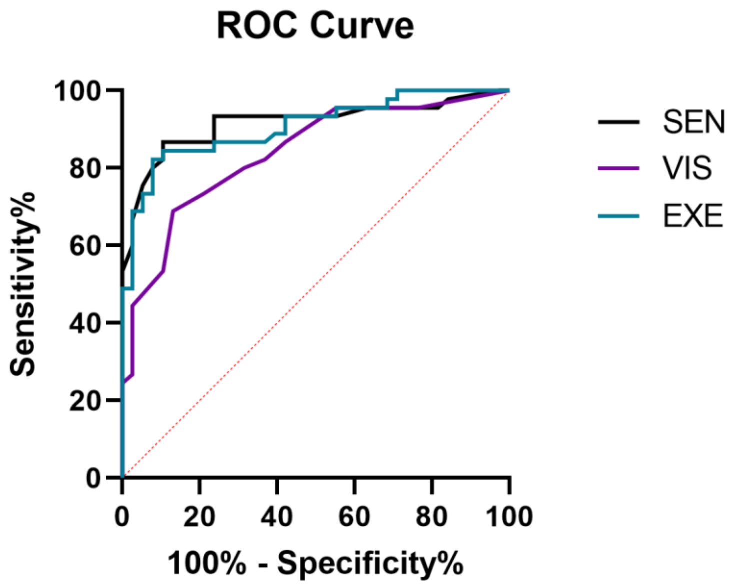

eReference

[1] Rami L, Mollica MA, García-Sanchez C, et al. The Subjective Cognitive Decline Questionnaire (SCD-Q): a validation study. Journal of Alzheimer's disease : JAD 2014;41.

[2] Levis B, Benedetti A, Thombs BD, Collaboration DESD. Accuracy of Patient Health Questionnaire-9 (PHQ-9) for screening to de.

[3] Burton JK, Stott DJ, McShane R, Noel-Storr AH, Swann-Price RS, Quinn TJ. Informant Questionnaire on Cognitive Decline in the Elderly (IQCODE) f.

[4] Rami L, Mollica M, García-Sanchez C, et al. The Subjective Cognitive Decline Questionnaire (SCD-Q): a validation study. Journal of Alzheimer's disease : JAD 2014;41.

[5] Kroenke K, Spitzer R, Williams J. The PHQ-9: validity of a brief depression severity measure. Journal of general internal medicine 2001;16.

[6] Jorm A, Scott R, Cullen J, MacKinnon A. Performance of the Informant Questionnaire on Cognitive Decline in the Elderly (IQCODE) as a screening test for dementia. Psychological medicine 1991;21.

[7] Boateng GO, Neilands TB, Frongillo EA, Melgar-Quiñonez HR, Young SL. Best Practices for Developing and Validating Scales for Health, Social, and Behavioral Research: A Primer. Frontiers in Public Health 2018;6.

[8] Raykov T, Marcoulides GA. Introduction to Psychometric Theory: Introduction to psychometric theory.

[9] Rousson V, Gasser T, Seifert B. Assessing intrarater, interrater and test-retest reliability of continuous measurements. Statistics in Medicine 2010;21.

[10] Kaiser HF, Rice J. Little Jiffy, Mark Iv. Educational and Psychological Measurement 2016;34.
